# Unveiling the Potential: Remote Monitoring and Telemedicine in Shaping the Future of Heart Failure Management

**DOI:** 10.3390/life14080936

**Published:** 2024-07-25

**Authors:** Ju-Chi Liu, Chun-Yao Cheng, Tzu-Hurng Cheng, Chen-Ning Liu, Jin-Jer Chen, Wen-Rui Hao

**Affiliations:** 1 Division of Cardiology, Department of Internal Medicine, Shuang Ho Hospital, Ministry of Health and Welfare, Taipei Medical University, New Taipei City 23561, Taiwan; liumdcv@s.tmu.edu.tw; 2 Division of Cardiology, Department of Internal Medicine, School of Medicine, College of Medicine, Taipei Medical University, Taipei City 11002, Taiwan; 3 Department of Medical Education, National Taiwan University Hospital, Taipei 100225, Taiwan; b05401143@ntu.edu.tw; 4 Department of Biochemistry, School of Medicine, College of Medicine, China Medical University, Taichung City 404333, Taiwan; thcheng@mail.cmu.edu.tw; 5 Center of Integrated, Shuang Ho Hospital, Ministry of Health and Welfare, Taipei Medical University, New Taipei City 23561, Taiwan; 12585@s.tmu.edu.tw; 6 Division of Cardiology, Department of Internal Medicine and Graduate Institute of Clinical Medical Science, China Medical University, Taichung 115201, Taiwan; jc8510@yahoo.com; 7 Institute of Biomedical Sciences, Academia Sinica, Taipei 11529, Taiwan

**Keywords:** heart failure (HF), remote monitoring, telemedicine, cardiovascular care, personalized medicine, patient engagement, treatment adherence, healthcare innovation, artificial intelligence (AI), machine learning, healthcare resource optimization

## Abstract

Heart failure (HF) remains a significant burden on global healthcare systems, necessitating innovative approaches for its management. This manuscript critically evaluates the role of remote monitoring and telemedicine in revolutionizing HF care delivery. Drawing upon a synthesis of current literature and clinical practices, it delineates the pivotal benefits, challenges, and personalized strategies associated with these technologies in HF management. The analysis highlights the potential of remote monitoring and telemedicine in facilitating timely interventions, enhancing patient engagement, and optimizing treatment adherence, thereby ameliorating clinical outcomes. However, technical intricacies, regulatory frameworks, and socioeconomic factors pose formidable hurdles to widespread adoption. The manuscript emphasizes the imperative of tailored interventions, leveraging advancements in artificial intelligence and machine learning, to address individual patient needs effectively. Looking forward, sustained innovation, interdisciplinary collaboration, and strategic investment are advocated to realize the transformative potential of remote monitoring and telemedicine in HF management, thereby advancing patient-centric care paradigms and optimizing healthcare resource allocation.

## 1. Introduction

Heart failure (HF) presents a significant global public health challenge characterized by inefficient blood pumping by the heart, resulting in debilitating symptoms and heightened mortality rates. The escalating burden of HF, driven by an aging populace and an upsurge in cardiovascular risk factors, underscores the necessity for innovative management strategies [1,2]. Remote monitoring and telemedicine have emerged as promising strategies to revolutionize HF care delivery, overcoming constraints in traditional healthcare frameworks. By harnessing telecommunications technology and digital health platforms, remote monitoring allows continuous surveillance of HF patients’ clinical parameters beyond conventional healthcare settings. Telemedicine facilitates real-time communication between patients and healthcare providers, enabling prompt intervention and personalized management [3].

This manuscript aims to elucidate the pivotal role of remote monitoring and telemedicine in reshaping HF management. By integrating current evidence and insights from clinical practice, it delineates the benefits, challenges, and personalized approaches associated with these innovative technologies, exploring their potential to enhance access to care, improve early detection of clinical deterioration, promote patient engagement, and optimize treatment outcomes among HF patients.

Moreover, recent advancements in machine learning (ML) have revolutionized healthcare, offering opportunities for personalized medicine and predictive analytics. Studies by Kao et al. (2023) [4] and Hsiu et al. (2022) [5] demonstrate ML applications in predicting atrial fibrillation risk and discriminating vascular aging, respectively, using electronic medical records and arterial pulse spectrum analysis. Similarly, research by Chen et al. (2022) [6] explores ML’s potential in understanding physiological responses to interventions, such as the side effects of COVID-19 vaccination. Furthermore, ML techniques have been crucial in hypertension management and cardiovascular risk assessment, as evidenced by studies like Liu et al. (2021) [7] and Lee et al. (2016) [8]. These investigations underscore ML’s utility in extracting meaningful insights from complex physiological data, aiding in early detection and personalized intervention strategies, particularly pertinent in HF management.

In the context of HF management, ML integration holds promise for enhancing risk stratification, optimizing treatment algorithms, and predicting clinical outcomes. Leveraging ML algorithms on remote monitoring data can enable early identification of subtle physiological changes indicative of HF exacerbations, empowering timely interventions and preemptive measures to prevent adverse events. Additionally, the manuscript scrutinizes the technical, regulatory, and socioeconomic hurdles impeding the widespread adoption of remote monitoring, telemedicine, and ML in HF care. It emphasizes tailored interventions and advanced technology integration to adeptly cater to individual patient exigencies. Ultimately, this manuscript aims to enrich the burgeoning literature on remote monitoring, telemedicine, and ML in HF management, advocating for sustained innovation, interdisciplinary collaboration, and strategic investment to unlock their transformative potential in advancing patient-centric care paradigms and optimizing healthcare resource allocation.

## 2. Evolution of Remote Monitoring and Telemedicine in Heart Failure

Historically, remote monitoring dates back to the early 20th century with telegraph and telephone-based consultations between physicians and patients. Breakthroughs in the latter half of the century, including portable medical devices and the internet, laid the groundwork for modern remote monitoring and telemedicine solutions, enabling real-time transmission of patient data and consultations [9,10].

In HF management, early remote monitoring efforts focused on transmitting electrocardiographic signals and ambulatory vital signs monitoring to detect arrhythmias and hemodynamic fluctuations. Over time, the integration of wireless technology, wearable sensors, and mobile applications expanded remote monitoring capabilities, enabling continuous monitoring of various physiological parameters, including fluid status, activity levels, and medication adherence [11]. Recent advancements in ML have further propelled the evolution of remote monitoring and telemedicine in HF management. Studies such as those by Kao et al. (2023) [4] and Hsiu et al. (2022) [5] demonstrate ML’s application in predicting atrial fibrillation risk and discriminating vascular aging, respectively, utilizing electronic medical records and arterial pulse spectrum analysis. Additionally, research by Chen et al. (2022) [6] highlights ML’s potential in understanding physiological responses to interventions, such as the side effects of COVID-19 vaccination. Furthermore, ML techniques have played a pivotal role in enhancing remote monitoring capabilities for hypertensive patients, as evidenced by studies like Liu et al. (2021) [7] and Lee et al. (2016) [8]. These investigations underscore ML’s utility in extracting meaningful insights from complex physiological data, aiding in early detection and personalized intervention strategies, particularly pertinent in HF management.

The adoption of remote monitoring and telemedicine in HF management has steadily grown, driven by the recognition of HF as a chronic condition requiring ongoing surveillance, advancements in healthcare informatics facilitating data integration, and the COVID-19 pandemic accelerating telemedicine adoption [12]. However, challenges persist, including technical issues, regulatory barriers, and disparities in access and digital literacy [13]. In summary, the evolution of remote monitoring and telemedicine, coupled with advancements in ML, has revolutionized HF management, offering opportunities for personalized, proactive care delivery. Addressing the remaining challenges is crucial to ensuring equitable access and maximizing benefits for all HF patients.

## 3. Benefits of Remote Monitoring and Telemedicine in Heart Failure Management

Remote monitoring and telemedicine have emerged as invaluable tools in HF management, offering benefits that enhance patient outcomes and optimize healthcare resource utilization (Table 1). Integrating recent literature enriches our understanding of these advantages and their implications for HF management. One significant advantage is the enhanced access to care they provide for HF patients. Enabling real-time monitoring of physiological parameters and symptoms from patients’ homes, remote monitoring reduces the need for frequent clinic visits, empowering patients to actively engage in their care [4]. This alleviates travel burdens and ensures timely intervention in cases of clinical deterioration. Studies show that remote monitoring interventions lead to earlier detection of worsening HF symptoms, enabling prompt adjustments to treatment regimens and reducing hospitalization risks [9,14].

Furthermore, remote monitoring and telemedicine foster patient engagement and self-management, which are pivotal to successful HF management. Through these platforms, patients proactively participate in self-care activities such as monitoring fluid intake, adhering to medication regimens, and adopting lifestyle modifications [15]. Recent advances in ML offer opportunities to enhance patient engagement by providing personalized insights and interventions based on real-time data analysis [6]. Moreover, the impact extends beyond individual patient outcomes to optimize broader healthcare resource utilization. By facilitating proactive HF management through early detection of clinical deterioration, remote monitoring reduces hospitalization frequency and duration, alleviating strain on healthcare systems and cutting costs [16]. Additionally, remote monitoring data guides more efficient resource allocation by identifying high-risk patients who may benefit from targeted interventions, maximizing healthcare resources’ value [17]. Insights from recent studies on ML applications in healthcare resource utilization underscore the potential for data-driven approaches to further optimize resource allocation and enhance healthcare efficiency [18,19]. In summary, remote monitoring and telemedicine offer benefits for HF management, including improved access to care, enhanced patient engagement and self-management, and optimized healthcare resource utilization. Enabling proactive, personalized care delivery, these technologies have the potential to transform HF management, improving outcomes for patients while relieving strain on healthcare systems. Continued investment in infrastructure, alongside efforts to address barriers, will be crucial to realizing their full potential in enhancing HF care delivery.

## 4. Challenges and Considerations

Despite the promising benefits of remote monitoring and telemedicine in HF management, several challenges and considerations must be addressed to realize their full potential and ensure equitable access and utilization (Table 1). Remote monitoring and telemedicine in the management of heart failure present a range of significant benefits and notable challenges. The primary benefits encompass improved patient outcomes through continuous health monitoring, which enables timely interventions and optimal management of the condition. Furthermore, telemedicine enhances patient engagement by providing convenient access to healthcare providers, thereby encouraging active participation in their own care. This approach also contributes to a reduction in hospital readmissions by allowing for early detection of symptoms and prompt management. Additionally, the cost-effectiveness of telemedicine is evident, as it reduces the need for in-person visits and hospital stays, ultimately lowering healthcare costs. Increased accessibility is another critical advantage, as telemedicine bridges the gap for patients residing in remote or underserved areas, ensuring they receive the necessary care.

Despite these benefits, several challenges persist. Technical issues such as the dependence on reliable internet connectivity and advanced technology can be significant barriers, particularly in rural areas with poor connectivity. Data privacy and security concerns are paramount, as handling sensitive patient information requires robust security measures to prevent breaches and maintain confidentiality. Patient compliance with the technology and adhering to medical advice are essential for successful remote monitoring. Health literacy poses another challenge, especially among the elderly, who may struggle with the technology required for telemedicine. Finally, the integration of telemedicine platforms with existing electronic health record systems can be complex and resource-intensive, posing an additional hurdle to widespread adoption.

One significant challenge is the array of technical issues that can impede the seamless operation of remote monitoring and telemedicine systems. Connectivity issues, such as unreliable internet connections or poor cellular coverage in rural areas, can disrupt data transmission and compromise the effectiveness of remote monitoring interventions [4]. Machine learning-based prediction and analysis offer opportunities for innovation in overcoming these barriers. Interoperability—the ability of different devices and systems to exchange and interpret data—remains a challenge, as many remote monitoring platforms operate in silos, leading to fragmented data and inefficiencies in care delivery. Additionally, ensuring robust data security measures to protect sensitive patient information from unauthorized access or cyberattacks is paramount to maintaining patient trust and compliance with privacy regulations [4,8].

Regulatory and reimbursement barriers pose significant problems for the widespread adoption of remote monitoring and telemedicine in HF management. While the COVID-19 pandemic prompted temporary regulatory changes and expanded reimbursement policies to facilitate telehealth services, many of these provisions are subject to revision or expiration [18]. Clarifying regulatory guidelines and establishing sustainable reimbursement models for remote monitoring and telemedicine services are critical to incentivizing healthcare organizations and providers to invest in these technologies and integrate them into routine clinical practice [18,20].

Addressing disparities in access to technology and digital literacy is another pressing concern. Socioeconomic factors, such as income level, education, and geographic location, can influence individuals’ ability to access and utilize remote monitoring and telemedicine services effectively. Vulnerable populations, including older adults, low-income individuals, and those residing in rural or underserved areas, may face barriers related to access to broadband internet, affordability of devices, and proficiency in using digital health tools [19]. Efforts to bridge the digital divide and promote digital literacy among diverse patient populations are essential to ensure equitable access to remote monitoring and telemedicine services and mitigate disparities in HF care outcomes [19,21].

Furthermore, patient and provider acceptance and adherence to remote monitoring protocols are critical determinants of success. While some patients may embrace the convenience and flexibility of remote monitoring, others may express concerns about privacy, data security, or the perceived loss of personal connection with their healthcare providers. Similarly, healthcare providers may encounter resistance or skepticism regarding the reliability and accuracy of remote monitoring data or the feasibility of integrating telemedicine into their workflow [8,22,23]. Addressing these concerns through education, training, and ongoing support is essential to foster acceptance and engagement among both patients and providers and promote sustained adherence to remote monitoring protocols [8,15,23].

To conclude, while remote monitoring and telemedicine hold significant promise for improving HF management, several challenges and considerations must be addressed to maximize their impact and ensure equitable access and utilization. By addressing technical, regulatory, socioeconomic, and behavioral barriers, stakeholders can work together to overcome these challenges and harness the full potential of remote monitoring and telemedicine to enhance HF care delivery and outcomes [4,18,19,22].

## 5. Personalized Approaches in Remote Monitoring and Telemedicine

Personalization is emerging as a key principle in remote monitoring and telemedicine for HF management, offering the potential to optimize care delivery and enhance patient outcomes (Table 2). Integrating insights from recent literature further enriches our understanding of these personalized approaches and their implications for HF management. Tailoring remote monitoring protocols based on individual patient characteristics and preferences is fundamental to personalized care. Considering factors such as age, comorbidities, disease severity, and technological proficiency, healthcare providers can design remote monitoring programs that meet the unique needs and preferences of each patient [24]. Insights from studies by Chen et al. (2022) [6] and Kao et al. (2023) [4] on machine learning-based prediction and analysis enable the customization of monitoring parameters and interventions tailored to individual patient profiles.

Furthermore, the integration of AI and ML algorithms holds promise for personalized risk stratification in HF management. AI-driven models can analyze large datasets encompassing clinical, physiological, and behavioral variables to predict individual patient trajectories and proactively intervene to mitigate adverse outcomes [25]. Leveraging insights from Hsiu et al. (2022) [5] on discriminating vascular aging using machine learning analysis, AI-powered decision support tools can assist healthcare providers in interpreting remote monitoring data and making informed clinical decisions tailored to each patient’s unique circumstances.

Customizing telemedicine interventions to meet the needs of diverse patient populations is another critical aspect of personalized care in HF management. Telemedicine platforms offer a range of modalities for remote communication, including video consultations, secure messaging, and remote monitoring applications [26]. By offering flexibility in communication channels and adapting communication styles to suit individual preferences and cultural backgrounds, healthcare providers can enhance patient engagement and satisfaction with telemedicine services. Insights from previous studies [19] can guide the customization of telemedicine interventions to accommodate patients with specific needs or limitations, ensuring equitable delivery of telemedicine services to all patients.

Overall, personalized approaches to remote monitoring and telemedicine are essential for optimizing HF management and delivering patient-centered care. By tailoring remote monitoring protocols, integrating AI-driven risk stratification models, and customizing telemedicine interventions to meet the diverse needs of patients, healthcare providers can enhance engagement, improve outcomes, and promote equity in access to care. Continued research, innovation, and collaboration are needed to further develop and implement personalized approaches in remote monitoring and telemedicine, ultimately advancing the field of HF management and improving the lives of patients affected by this chronic condition.

## 6. Future Directions and Implications

The future of remote monitoring and telemedicine in HF management holds significant promise, with ongoing innovations poised to transform healthcare delivery models, enhance patient outcomes, and optimize resource utilization. Integrating insights from recent literature further enriches our understanding of potential future directions and implications of remote monitoring and telemedicine in HF management.

Innovations in remote monitoring technology and telemedicine platforms are expected to drive significant advancements in HF management. The integration of wearable sensors, implantable devices, and Internet of Things (IoT) technologies enables continuous monitoring of physiological parameters relevant to HF progression, such as biomarkers, fluid status, and physical activity levels [27]. These advancements offer opportunities for comprehensive monitoring, facilitating earlier detection of HF exacerbations and timely interventions.

Furthermore, advancements in remote monitoring platforms, such as cloud-based data analytics, AI, and ML, facilitate real-time analysis of patient data and predictive modeling. AI-driven models predict individual patient trajectories and enable personalized interventions tailored to unique circumstances [28]. Leveraging insights from machine learning analyses [5,6], these platforms optimize treatment strategies and enhance patient outcomes through proactive and personalized care delivery.

The potential impact of remote monitoring and telemedicine on healthcare delivery models, patient outcomes, and resource utilization is significant. By enabling remote access to healthcare services and reducing the need for in-person visits, these technologies improve access to care, enhance patient engagement, and optimize treatment outcomes for HF patients [29]. Moreover, by empowering patients to participate in their care and providing healthcare providers with real-time data and decision-support tools, these technologies facilitate more efficient and effective care delivery, ultimately reducing healthcare costs and maximizing resource value [30].

Opportunities for research, collaboration, and overcoming barriers to implementation are essential for realizing the full potential of remote monitoring and telemedicine in HF management. Continued investment in research and development is required to refine and validate remote monitoring technologies, evaluate their impact on patient outcomes, and identify strategies for integrating them into routine clinical practice [31]. Collaboration among stakeholders, including healthcare providers, technology developers, policymakers, and patient advocacy groups, is critical for addressing regulatory, reimbursement, and interoperability challenges, fostering innovation, and driving widespread adoption of these solutions [32].

Efforts to promote digital literacy, address healthcare disparities, and ensure equitable access to remote monitoring and telemedicine services are essential for realizing the promise of these technologies in improving HF care delivery and outcomes for all patients [33]. By addressing these challenges and harnessing the full potential of remote monitoring and telemedicine, stakeholders can advance the field of HF management and improve the lives of patients affected by this chronic condition.

There are some limitations. Given that this is not a systematic review, it inherently possesses certain weaknesses and selection biases that are challenging to mitigate. Additionally, remote monitoring and telemedicine prove to be less practical for patients with hearing or visual impairments or those with lower cultural or intellectual levels. These limitations underscore the need for tailored approaches to ensure equitable access and effectiveness in digital health interventions.

## 7. Conclusions

In conclusion, remote monitoring and telemedicine have emerged as indispensable tools in the management of HF, offering benefits including improved access to care, enhanced patient engagement, and optimized resource utilization (Table 3). Despite challenges, the potential impact of remote monitoring and telemedicine on healthcare delivery models and patient outcomes is profound.

Continued investment, innovation, and collaboration are essential to harnessing the full potential of remote monitoring and telemedicine in HF management. Insights from recent literature, such as machine learning-based prediction models [4] and analyses revealing distinct arterial pulse variability [5,6], highlight the importance of leveraging technological advancements to improve HF care delivery.

Efforts to promote digital literacy, address healthcare disparities, and ensure equitable access to remote monitoring and telemedicine services are critical for realizing the promise of these technologies in improving HF care delivery and outcomes for all patients. Collaborative initiatives, informed by population-based studies [19], can drive policy changes and resource allocation strategies to address these challenges.

In summary, remote monitoring and telemedicine represent transformative opportunities to advance the field of HF management and improve the lives of patients affected by this chronic condition. By embracing innovation, collaboration, and patient-centered approaches, we can push the boundaries of HF care delivery and achieve better outcomes for patients worldwide. Insights from studies on healthcare resource utilization [19] and the impact of sleep quality on quality of life in HF patients [22] underscore the multidimensional nature of HF management and the importance of holistic approaches in optimizing patient care.

Through ongoing research, interdisciplinary collaboration, and a commitment to addressing healthcare disparities, we can realize the full potential of remote monitoring and telemedicine in HF management, paving the way for more effective, accessible, and patient-centered care delivery.

## Figures and Tables

**Table 1:** Key benefits and challenges of remote monitoring and telemedicine in heart failure management.

Aspect	Description
Benefits	
Improved Patient Outcomes	Remote monitoring allows for continuous tracking of patient health, leading to timely interventions and better management of heart failure.
Enhanced Patient Engagement	Telemedicine provides patients with easy access to healthcare providers, encouraging active participation in their own care.
Reduced Hospital Readmissions	Early detection of symptoms and prompt management via telemedicine can prevent hospital readmissions, which are common in heart failure patients.
Cost-Effectiveness	By reducing the need for in-person visits and hospital stays, remote monitoring and telemedicine can lower healthcare costs.
Increased Accessibility	Telemedicine bridges the gap for patients in remote or underserved areas, ensuring they receive necessary care.
**Challenges**	
Technical Issues	Dependence on reliable internet and technology can be a barrier, especially in rural areas with poor connectivity.
Data Privacy and Security	Handling sensitive patient data requires robust security measures to prevent breaches and ensure confidentiality.
Patient Compliance	Successful remote monitoring relies on patients’ adherence to using the technology and following medical advice.
Health Literacy	Some patients, particularly the elderly, may struggle with the technology required for remote monitoring and telemedicine.
Integration with Existing Systems	Seamless integration of telemedicine platforms with existing electronic health record systems can be complex and resource-intensive.

This table provides a concise overview of both the advantages and the potential obstacles associated with the use of remote monitoring and telemedicine in managing heart failure.

**Table 2:** Types of remote monitoring technologies and their applications in heart failure management.

Technology	Description	Applications
Wearable Devices	Devices such as smartwatches and fitness trackers that monitor vital signs like heart rate, activity levels, and sleep patterns.	Continuous monitoring of heart rate, physical activity, and sleep quality to detect early signs of heart failure exacerbation.
Implantable Devices	Devices like cardiac resynchronization therapy (CRT) and implantable cardioverter defibrillators (ICD) that provide real-time monitoring and therapeutic intervention.	Monitoring of cardiac function and automatic delivery of therapy to manage arrhythmias and other cardiac events.
Remote Monitoring Platforms	Comprehensive systems that collect and analyze data from various sources, including wearables and implantable devices.	Integration and analysis of patient data to provide holistic insights and enable proactive management of heart failure.
Mobile Health Apps	Smartphone applications designed to track symptoms, medication adherence, and lifestyle factors such as diet and exercise.	Facilitating patient self-management, education, and communication with healthcare providers.
Telemedicine Platforms	Online systems that enable virtual consultations, remote check-ins, and real-time communication between patients and healthcare providers.	Providing accessible healthcare services, routine check-ups, and emergency consultations without the need for in-person visits.
Home-Based Diagnostic Tools	Devices such as digital blood pressure monitors, weight scales, and ECG monitors that patients use at home to track their health metrics.	Daily monitoring of vital signs and early detection of health changes, allowing for timely medical intervention.
Artificial Intelligence and Analytics	Advanced software that uses machine learning algorithms to predict patient outcomes and personalize treatment plans based on collected data.	Enhancing decision-making processes for healthcare providers by predicting disease progression and optimizing treatment strategies.

This table outlines various remote monitoring technologies, their descriptions, and specific applications in the context of heart failure management, providing a clear overview of the tools available and their practical uses.

**Table 3:** Comparison of traditional in-person heart failure management and remote monitoring/telemedicine approaches.

Aspect	Traditional In-Person Management	Remote Monitoring/Telemedicine
Accessibility	Limited to geographic location and availability of healthcare providers.	Accessible from anywhere with internet connectivity, bridging gaps for remote or underserved areas.
Frequency of Monitoring	Periodic check-ups, typically scheduled weeks or months apart.	Continuous or frequent monitoring, allowing for real-time data collection and timely interventions.
Patient Engagement	Patient engagement often limited to scheduled visits; may be passive between appointments.	Encourages active patient participation through regular updates, use of apps, and constant feedback loops.
Response Time	Potential delays in response to symptoms or health changes, depending on appointment availability.	Rapid response to changes in patient condition, enabling prompt medical attention and adjustments in treatment.
Cost	Costs include travel, time off work, and potential hospital admissions for exacerbations.	Reduces overall costs by minimizing travel, preventing hospital readmissions, and facilitating efficient care.
Data Collection	Limited data collected during in-person visits, often relying on patient recall and periodic testing.	Comprehensive data collection from multiple sources (wearables, apps, devices) providing a more complete health picture.
Care Coordination	Coordination can be fragmented, with different providers handling various aspects of care.	Integrated platforms can streamline communication and coordination among healthcare providers.
Health Outcomes	Dependent on the frequency of visits and patient’s ability to seek timely care.	Potentially improved outcomes through early detection, continuous monitoring, and personalized interventions.
Patient Convenience	Involves travel, waiting times, and possible disruptions to daily routines.	Offers convenience with virtual consultations and home-based monitoring, reducing the need for frequent clinic visits.
Technology Dependence	Minimal technology required; primary reliance on face-to-face interactions and physical examinations.	Relies heavily on technology, requiring patients to use devices, apps, and ensure internet connectivity.

This table provides a side-by-side comparison of traditional in-person heart failure management versus remote monitoring and telemedicine, highlighting the differences in accessibility, frequency of monitoring, patient engagement, response time, cost, data collection, care coordination, health outcomes, patient convenience, and technology dependence.
